# Seasonal Dynamics of the Plant Community and Soil Seed Bank along a Successional Gradient in a Subalpine Meadow on the Tibetan Plateau

**DOI:** 10.1371/journal.pone.0080220

**Published:** 2013-11-11

**Authors:** Miaojun Ma, Xianhui Zhou, Wei Qi, Kun Liu, Peng Jia, Guozhen Du

**Affiliations:** State Key Laboratory of Grassland and Agro-Ecosystems, School of Life Science, Lanzhou University, Lanzhou, P. R. China; University of Rome 'La Sapienza', Italy

## Abstract

**Background:**

Knowledge about how change the importance of soil seed bank and relationship between seed mass and abundance during vegetation succession is crucial for understanding vegetation dynamics. Many studies have been conducted, but their ecological mechanisms of community assembly are not fully understood.

**Methodology:**

We examined the seasonal dynamics of the vegetation and soil seed bank as well as seed size distribution along a successional gradient. We also explored the potential role of the soil seed bank in plant community regeneration, the relationship between seed mass and species abundance, and the relative importance of deterministic and stochastic processes along a successional gradient.

**Principal Findings:**

Species richness of seed bank increased (shallow layer and the total) and seed density decreased (each layer and the total) significantly with succession. Species richness and seed density differed significantly between different seasons and among soil depths. Seed mass showed a significant negative relationship with relative abundance in the earliest successional stage, but the relationships were not significant in later stages. Seed mass showed no relationship with relative abundance in the whole successional series in seed bank. Results were similar for both July 2005 and April 2006.

**Conclusions:**

The seed mass and abundance relationship was determined by a complex interaction between small and larger seeded species and environmental factors. Both stochastic processes and deterministic processes were important determinants of the structure of the earliest stage. The importance of seed bank decreased with succession. The restoration of abandoned farmed and grazed meadows to the species-rich subalpine meadow in Tibetan Plateau can be successfully achieved from the soil seed bank. However, at least 20 years are required to fully restore an abandoned agricultural meadow to a natural mature subalpine meadow.

## Introduction

Knowledge of how the species composition of the soil seed bank varies within and across spatial and temporal scales is crucial for fully understanding vegetation dynamics and mechanisms [1]–[2]. Unfortunately, the dynamics of subalpine soil seed banks have received comparatively little attention to other ecosystems, and there are no studies of changes during succession in subalpine meadows.

In other grassland ecosystems, early successional species produce high densities of persistent seeds that are preserved in the soil even after a respective species disappears from the aboveground vegetation [3]. Therefore, the seed bank is dominated by early successional species in later stages [4]–[6]. In such situations, the seed bank remains unvaried regardless of aboveground vegetation change and represents a storehouse (inflow) during succession [3]. However, an alternative hypothesis suggests that early successional species disappear from the seed bank when the species disappear from the vegetation, and seeds of later successional species gradually become incorporated into the soil [7]. In the latter case, the species composition of the seed bank changes as the aboveground vegetation changes, and seed banks represent a spillover (outflow) during the successional series. Whether the soil seed bank functions as a storehouse or a spillover change during succession is the main question addressed by this study.

How plant communities are established and maintained over time is an important topic in plant ecology [8]. Study of the species composition similarity between the seed bank and vegetation during succession can increase our mechanistic understanding of community assembly [9] and provide appropriate management methods [10]. However, [11] found that the regeneration of vegetation cannot rely on the seed bank, and persistent seeds were not the key mechanism of species resilience [9]. In cold habitats, seed banks have a minor role compared to vegetative growth/clonal growth in regeneration [12]–[13]. Very little work has addressed how the vegetation and the seed bank resemble each other during secondary succession [10], [14] or the role of seed banks to regeneration of plant communities [15]. We know of none for subalpine meadow succession.

For a given size and reproductive allocation, a species must trade off the advantages of dividing its limited resources into many small seeds or producing fewer large seeds [16]–[17]. Research has shown that seed mass is highly correlated with plant abundance [18]. According to a successional model proposed to examine the relationship between seed mass and the abundance in plant communities [18], there are numerous small-seeded species in early successional stages that are good colonisers with high dispersal capacities related to high seed production [18]-[20]. However, as succession progresses, there are fewer but larger-seeded species dominated the plant communities [18], [20]–[23]. These species have higher survival and competitive ability because of larger seed reserves. Hence, the difference in seed allocation strategies along the successional gradient could be summarized as a colonization-competition tradeoff. If so, there would be a negative correlation between seed mass and abundance in early successional stages, and this would gradually decrease and disappear in mid successional stages. Eventually, a positive correlation would develop. However, [24] found no significant relationship between seed size and abundance in northern England and may be explained by different responses of small and large-seeded species to environmental variation. Although many studies [18], [25]–[26] have theoretically evaluated such predictions, very few empirical studies have been conducted to test them [20], especially in alpine and subalpine meadows and never on the Tibetan Plateau. In addition, so far no studies exist of seed mass change in the seed bank along a successional gradient.

The main objectives of the study were to answer the following questions:

1. Is there a relationship between seed mass and abundance in plant communities along a successional gradient?
2. What is the potential contribution of the transient and persistent seed bank to plant community regeneration along a successional gradient.

## Methods

### Ethics Statement

No permits were required to carry out this study. The owner of all the study sites is PR.China. The Chinese government give us permission to conduct the study on these sites. We confirm that the field studies are in this did not involve endangered or protected species. No vertebrate studies in this manuscript.

### Study sites

The study was carried out in a subalpine meadow of the eastern Tibetan Plateau, Hezuo, Gansu province, China (N34°55′, E102°53′), at an elevation of 2,900-3,000 m asl. The average temperature is 2.4°C, varying from from −9.9°C in January to 12.8°C in July, and the precipitation is 531.6 mm per year, varying from 2.4 mm in January to 110.3 mm in July. The vegetation of the mature meadows is characterised by *Poa* spp, *Agrostis hugoniana*, *Festuca ovina*, *Elymus nutans*, *Stipa aliena*, *Kobresia humilis*, *Gentiana macrophylla, Aster flaccidus,* and *Ligularia virgaurea* (in order of dominance) [5]. The soil type was an alpine meadow soil [5]. The germination experiment was conducted in the Research Station of Alpine Meadow and Wetland Ecosystems of Lanzhou University (Hezuo Branch Station), Gansu, China, also on the eastern Tibetan plateau, at an elevation of 2900 m above sea level. The average temperature there is 2.0°C and precipitation is 557.8 mm.

The subalpine meadows at Hezuo have areas with different agricultural use histories that provide a good place to study successional dynamics of vegetation and seed bank during secondary succession. We used a space for time substitution using a chronosequence [1], [5]. Seed bank sampling and assessments of vegetation composition were carried out at four successional meadows: three abandoned stages (1-yr, 10-yr, and 20-yr), once used for cultivated crops (*Hordeum vulgare var*. nudum) have been abandoned for different lengths of time [5], and a typical subalpine meadow, which had not been cultivated or grazed (Table 1).

**Table 1:** Descriptions of the habitat types investigated.

Successional age	Dominant species	Description
1-yr	*Artemisia hedinii*, *Aconitum gymnandrum*, *Plantago asiatica*, and *Potentilla anserine*.	The meadows in 1-yr stage were abandoned from intensive cultivation only 1 year. The vegetation cover is low. Grazing and human disturbance were prohibited after it was abandoned. Annual ruderals dominate the vegetation. There are many gaps, which were created by Tibetan Pika (*Ochotona curzoniae*), marmot (*Marmota himalayana*), in this habitat.
10-yr	*Elephantopus mollis*, *Elymus dahuricus*, *Roegneria nutans*, and *Medicago ruthenica*.	The meadows were abandoned from arable activity for nearly 10 years, and the differences with 1-yr habitat are now quite significant. Perennial graminoids have established dominance and vegetation is taller than other meadows. The abundance of annual ruderals from 1-yr habitat has declined. The meadow has been lightly grazed by livestock (e.g. Tibetan sheep and yak) since abandonment.
20-yr	*Kobresia humilis*, *Artemisia tangutica*, and *Elymus dahuricus*.	The meadows were abandoned from agriculture activity 20 years. The meadow has been managed with a light and intermittent grazing regime by domestic animals (e.g. Tibetan sheep and yak) for 20 years, and the vegetation is species rich. There was no significant difference with mature meadow after 20 years restoration.
Mature meadow (undisturbed)	*Thalictrum alpinum*, *Kobresia humilis*, *Scirpus pumilus*, and *Stipa capillata*.	Never cultivated, a typical mature subalpine meadows in the eastern Tibetan Plateau. The vegetation is very species rich where it is covered with grasses and sedges. The mature meadows may be lightly grazed by livestock (e.g., yak and Tibetan sheep).

All habitats have same slope, exposure, altitude, annual mean temperature (2.4°C) and annual mean precipitation (531.6 mm).

### Soil seed bank sampling

Soil samples were collected: in July 2005, after the spring germination flush but before dispersal of the current season seeds, to assess the persistent seed bank; and in early April 2006 before field seed germination, to capture both the transient and the persistent seed bank. The total area of each successional stage meadows was at least 120 ha [27]. The agriculture land in eastern Tibetan Plateau shows scattered distribution because of the varied topography, and there are many small size patches (900 m^2^–3000 m^2^) in each successional stage. Ten randomly selected replicate sites (10 m×10 m, July 2005; 20 m×20 m, April 2006) were established in different patches of each successional stage, and each site was isolated in every single randomly selected patch. The sites are far apart from each other (100–300 m between any two sites). All sites had the same slope and exposure. Each site could as an independent spatial replicate for the each successional age. In each of 10 plots (0.4 m×1 m, July 2005; 5 m×5 m, April 2006), randomly distributed in each site, 10 cylindrical soil cores (3.6 cm diameter) were collected randomly. The soil cores were separated into three layers, shallow (0–2 cm deep), mid (2–7 cm deep), and deep (7–12 cm deep). Ten cores from each depth were pooled for each plot. Overall, there were 30 samples from each site (10 samples in each layer), and 300 soil samples for each meadow, and 1200 soil samples in all. Thus, the area sampled at each meadow was 1.02 m^2^, and a total bulk of soil samples of 0.151 m^3^.

### Treatment of samples and maintenance of seed trays

Soil samples were stored for 15 days until they could be processed. The seed bank was sampled by the concentration method followed by seedling emergence [5]–[6], [28]–[31]. Samples were first sieved to remove coarse debris and root fragments, after which they were washed and sieved through a coarse (4 mm mesh) and then a fine (0.2 mm mesh) sieve. The concentrated samples were spread evenly on a layer of sterile sand (sterilized at 140°C for 24 h) (depth 15 cm) in sterile plastic germination trays (width 30 cm). Thirty control trays with only sterilized sand were set alongside the experimental trays to determine the presence of airborne seeds. Trays were watered regularly. Once identified, seedlings were removed to keep densities low, and those that could not be identified immediately were grown separately until identification was possible. After 5 months when no more seedlings emerged for several consecutive weeks, sampling was stopped. Subsequent sifting and careful inspection found that no seeds remained.

### Seed mass determination

Seeds of 140 out of the 169 species found in the vegetation and seed bank were collected from August to October in 2005 and 2006 at the start of natural dispersal. The seeds were air-dried before being weighed. Seeds of a given species were pooled, well mixed, and three subsamples of 100 seeds selected. Seed mass was defined as the mass of the embryo and endosperm, plus the seed coat; further dispersal structures (e.g. hairs) were not included. The average mass of the three subsamples was used as the seed mass variable [29].

### Above-ground vegetation sampling

Vegetation sampling was performed at the peak of the summer growing season (July, 2005 and 2006), using 50 cm×50 cm-sized quadrats placed within each of the four successional meadows where soil samples had been collected. Ten quadrats were sampled in each successional stage in 2005 and five quadrats in 2006. We recorded the presence and cover of all species within each quadrat. Cover values (percentage cover) were estimated using the Braun-Blanquet scale [32].

### Data analysis

The species richness, cover, functional group (Grass, sedge and forbs) cover of vegetation and seed mass of the four successional stages were compared using one-way analysis of variance (ANOVA) and the Tukey test; data for each season were analyzed separately. Seed density and species richness of the seed bank of the successional stages were compared using ANOVA and the Tukey test; data for each depth (0–2 cm, 2–7 cm, 7–12 cm) and the whole (0–12 cm) were analyzed separately. Prior to analysis, data were examined for normality and homogeneity of variance, and data were transformed. Species richness in aboveground vegetation was analysed using a repeated measures ANOVA to test for general effects and their interactions, with year (2005 and 2006) as within-subjects effect and successional stage (1-yr, 10-yr, 20-yr and mature meadow) as between-subjects effects. Seed bank density and species richness in soil seed bank were analysed a repeated-measures ANOVA to test for general effects and their interactions, with soil depth (0–2 cm, 2–7 cm and 7–12 cm) and season (July, 2005 and April, 2006) as within-subjects effect and successional stage (1-yr, 10-yr, 20-yr and mature meadow) as between subjects effects. All ANOVA tests were conducted with a SPSS 13.0 program. We examined the relationships between relative abundance and seed mass during succession by calculating Kendall's tau-b correlation coefficients. Vegetation and seed bank data for in two seasons were analyzed separately. Ordination by non-metric multidimensional scaling (NMDS) was used to evaluate species composition similarity among successional stages for seed banks and vegetation in two seasons (July, 2005; May, 2006). Data for both seasons were analyzed together. Ordination was made using the R-program for Windows version 2.0.7, applying package VEGAN by Jari Oksanen.

## Results

### Plant community changes

**Figure 1:**
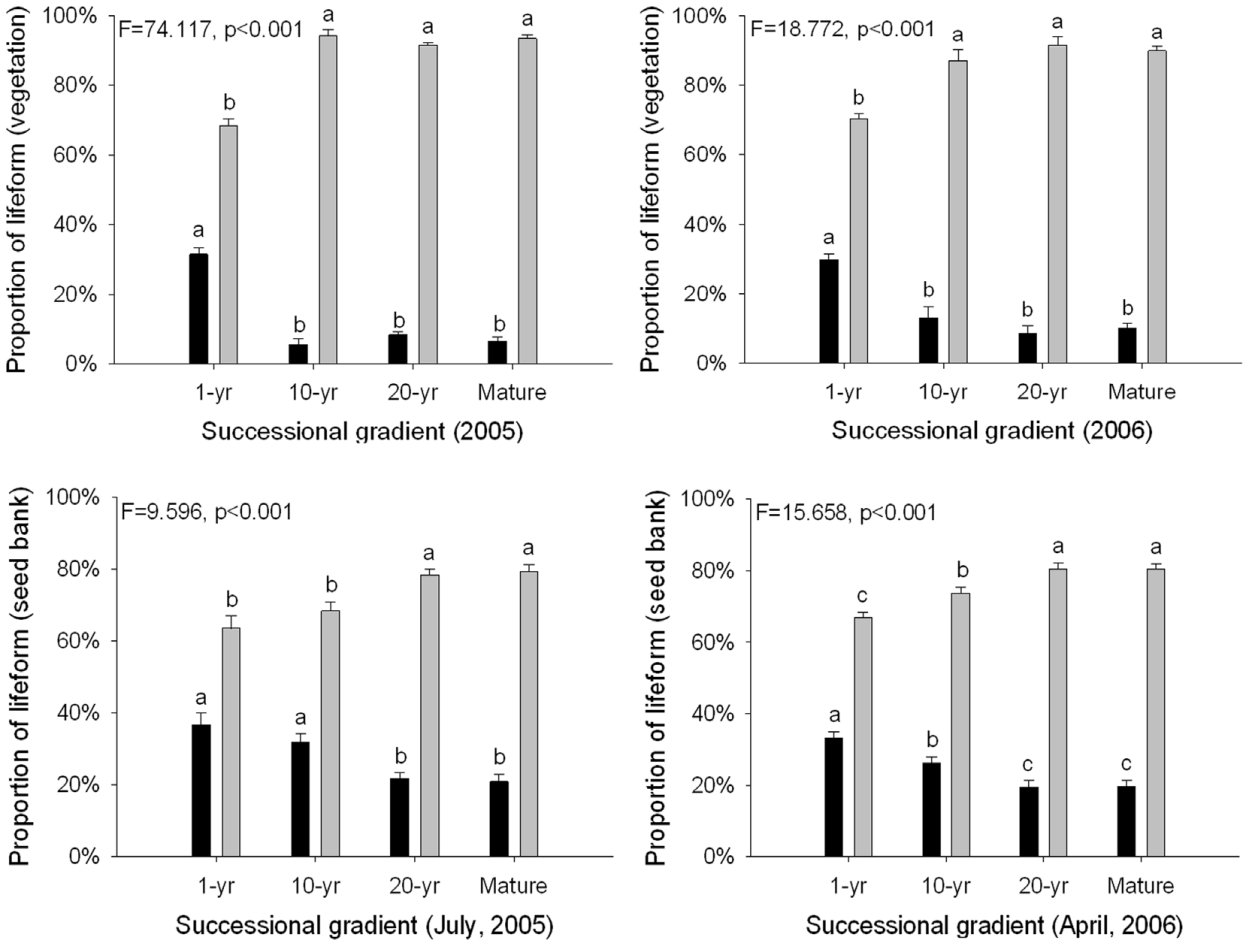
The proportion of life forms changes (per quadrat, mean±SE, n = 10. Black bar: annual and biennial species, gray bar: perennial species) in vegetation (top) and seed bank (bottom) in different seasons (June – left; May –right) along a disturbance gradient in the Tibetan Plateau. Letters indicate significant differences (ANOVA, Tukey range test) of mean values of disturbance type of a date.

Altogether 101 species, belonging to 26 families were recorded in both the seed bank and vegetation in 2005. Of these, 14.9% were annuals, 5% biennials, and 80.1% perennial herbs. In 2006, 95 species were recorded, belonging to 20 families. Of these, 14.4% were annuals, 5.6% biennials, and 80% perennial herbs. The proportion of perennial species increased along the successional gradient both in 2005 and in 2006 (Fig. 1).

**Figure 2:**
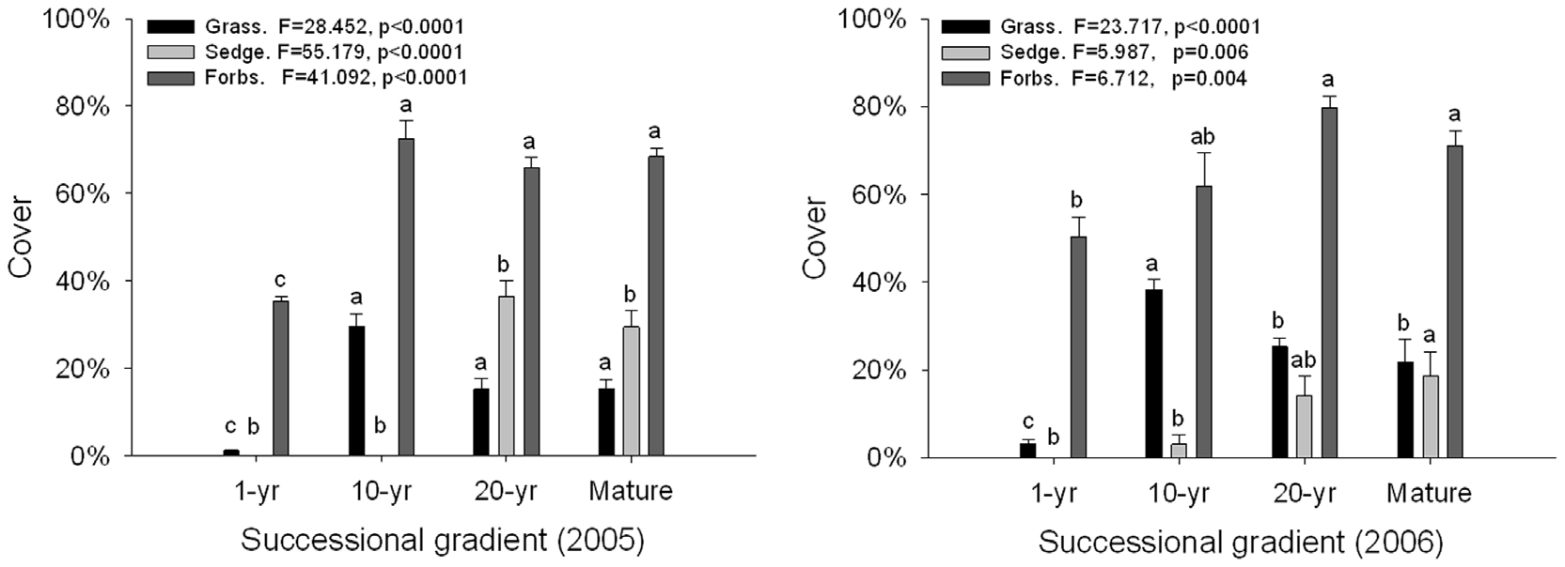
Comparison of functional group vegetation cover (per quadrat, mean±SE) along a succession gradient in the subalpine meadow of Tibetan Plateau. Data are for 2005 and 2006; functional groups are grasses, sedges, and forbs. Letters indicate significant differences (ANOVA, Tukey range test) of mean values.

The cover of functional groups differed significantly both in 2005 and in 2006 (Fig. 2). The lowest cover of grasses and sedges appeared in the 1-yr meadow, and increased as succession progressed. Grass cover increased after 10 yr abandonment and then decreased slightly in later stages. Sedge cover increased significantly after 20 yr abandonment.

**Figure 3:**
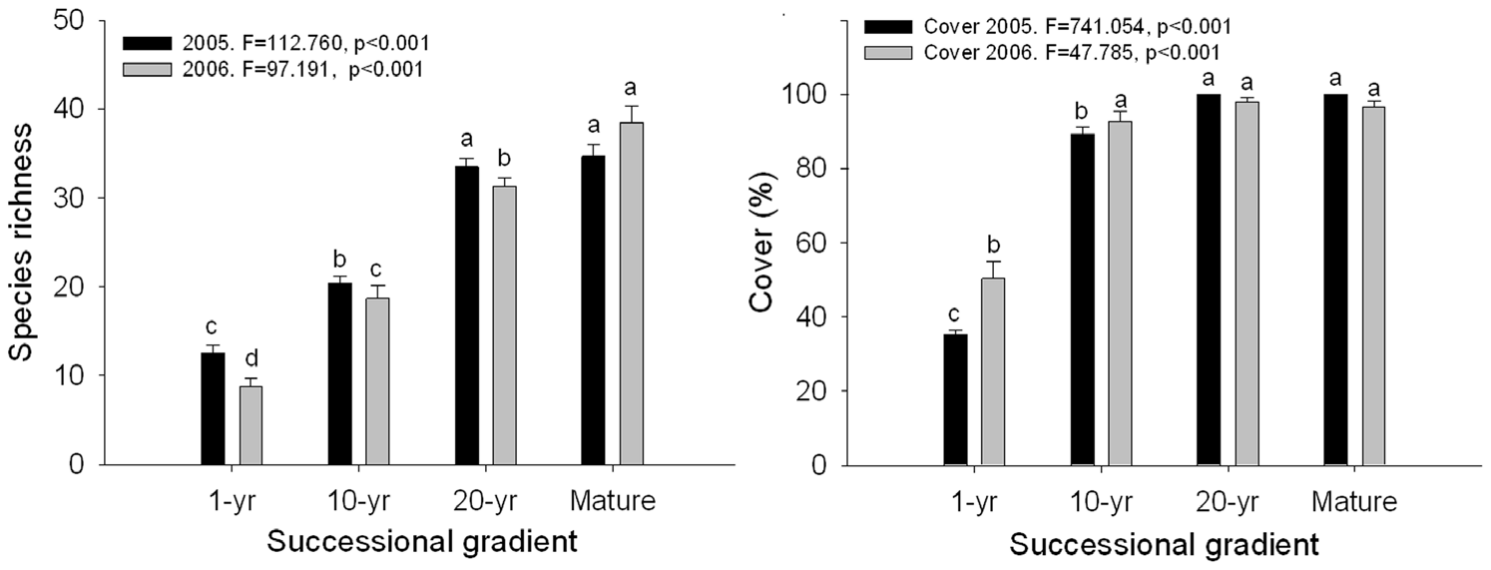
Species richness (left) and cover (right) (per quadrat, mean±SE) changes in vegetation along a successional gradient in the subalpine meadow of Tibetan Plateau. Letters indicate significant differences (ANOVA, Tukey range test).

Species richness per quadrat increased significantly along a successional gradient both in 2005 and 2006 (Fig. 3). Results from both 2005 and 2006 were similar (Table. 2; Fig. 3) and there was no interaction between successional stage and year. There was no significant difference between the 20-yr meadow and Mature meadow, or among 10-yr, 20 yr and Mature meadows in 2006.

**Table 2:** Repeated measure ANOVA (GLM) for the effect of year (repeated facor) and successional stage (non-repeated factor) on species richness in aboveground vegetation, and effect of season, depth (repeated facor) and successional stage (non-repeated factor) on species richness and seed density in soil seed bank.

		Seed density		Species richness	
Variable	df	F	*P*	F	*P*
**Aboveground vegetation**					
year	1			0.992	0.334
Year×successional stage	3			0.352	0.788
Error(year)	16				
**Soil seed bank**					
Depth	2	11.754	**0.0001**	88.962	**0.0001**
Soil depth×successional stage	6	24.506	**0.0001**	21.525	**0.0001**
Error (depth)	72				
Season	1	391.274	**0.0001**	534.193	**0.0001**
Season×successional stage	3	2.363	0.087	26.330	**0.0001**
Error (season)	36				
Soil depth×season	2	26.064	**0.0001**	226.458	**0.0001**
Soil depth×season×successional stage	6	23.834	**0.0001**	41.430	**0.0001**
Error (season*depth)	72				

Seed density comparisons were carried out using log transformed data. Differences shown in bold are statistically significant.

**Figure 4:**
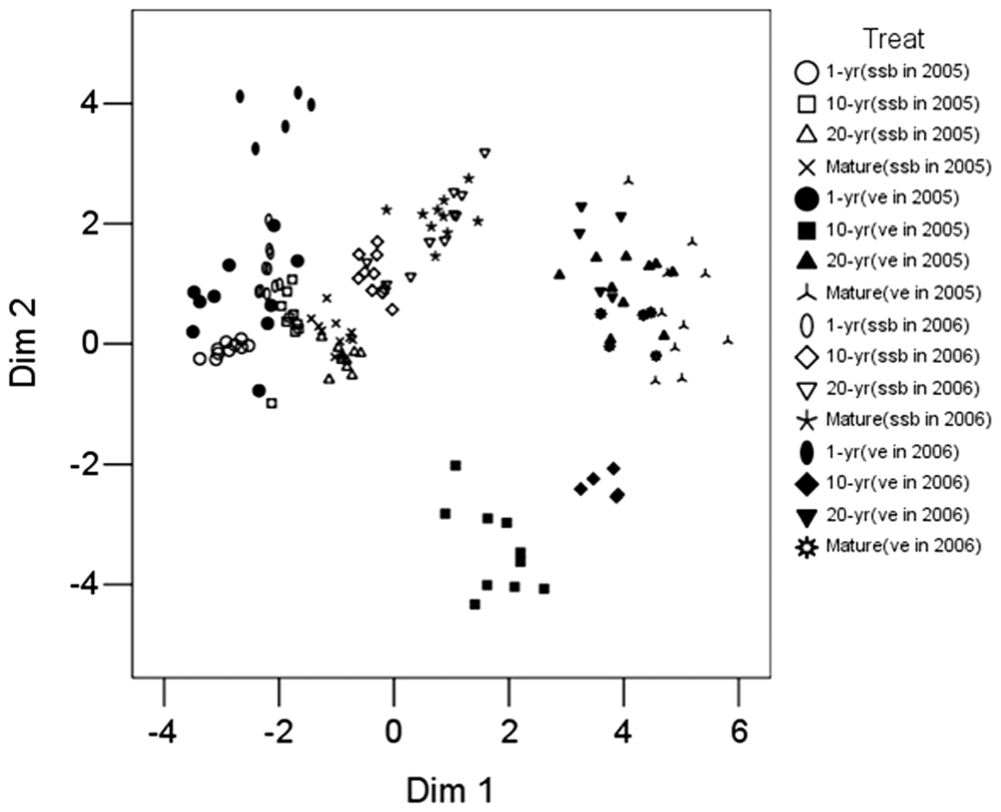
Two-dimensional nonmetric multidimensional scaling (NMDS) ordination of species composition of seed banks and vegetation during succession in different seasons in a subalpine meadows of the Tibetan Plateau (Stress value = 0.2). Ordination was based on relative abundance data. Different marks represent different seed bank and vegetation types. There were 10 samples for each seed bank, and 10 vegetation plots in 2005 and 5 plots in 2006. The location of ordination points within each diagram indicates the degree of similarity between each one.

The aboveground vegetation showed a clear segregation from the first and second Dim, whereas the 20-yr and Mature meadows overlapped (Fig. 4). There was little difference in four successional meadows between 2005 and 2006 (Fig. 4). There was a successional trend of 1-yr habitat generally becoming more similar to Mature meadow with time-since-abandonment, although they remained floristically distinct.

### Seed bank changes

Visual inspection of samples after germination revealed no seeds remained, indicating an accurate estimation of the seed bank. No seedlings were recorded in the control trays.

Overall, 113 species emerged from the two seasonal sets of seed bank samples. Two species could only be identified to the family level (Asteraceae sp. and Ranunculaceae sp.), two only to genus (*Cirsium* sp. and *Pedicularis* sp), and two were unknown species.

Altogether 18464 seedlings representing 64 species emerged from July, 2005 soil samples; 23.4% were annuals, 4.7% biennials, and 71.9% perennial herbs. Altogether 20423 seedlings from 98 species were recorded from May, 2006 samples; 23.4% were annuals, 3.2% biennials, and 73.4% perennial herbs. The proportion of perennial species in the seed bank showed an increasing trend with succession regardless of season (Fig. 1).

**Figure 5:**
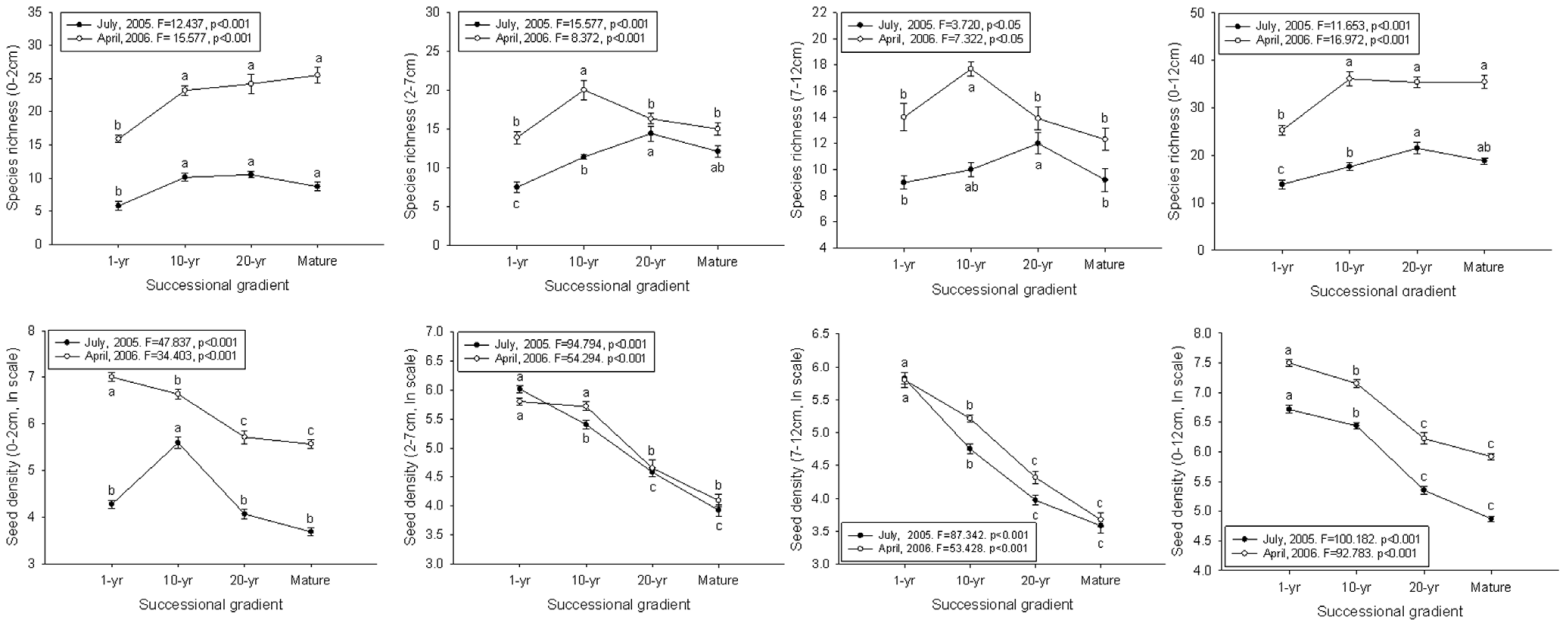
Mean species richness (per plot) (top row) and seed density m^−2^ (±SE) (bottom row) (±SE) at four soil depths (0–2 cm, 2–7 cm, 7–12 cm and 0–12 cm)classes along a succession gradient on a subalpine meadow of Tibetan Plateau. Letters indicate significant differences (ANOVA, Tukey range test) among mean values between successional stages for a given date disturbance type of a date.

Species richness and seed density per sample differed significantly along the successional gradient for each layer (0–2 cm, 2–7 cm and 7–12 cm) and for total (0–12 cm) (Fig.5). Species richness showed an increase trend with succession in the shallow layer (0–2 cm) and total (0–12 cm), and no trend in mid (2–7 cm) and lowest layer (7–12 cm) both in July 2005 and April 2006, while seed density decreased significantly during vegetation succession in each soil layer (Fig. 5). Species richness and seed density per sample differed significantly between seasons and among soil depths (Table. 2, Fig. 5).

For species richness, there were significant interactions between successional stage, season, and depth (Table 2). For seed density, all interactions were significant except between season—successional stage (Table. 2).

For July 2005 data, the 1-yr group has a little difference in Dim 1 from 10-yr, 20-yr and Mature meadow groups, which clustered together. In April, 2006, 1-yr, 10-yr and Mature meadow groups were separated by Dim 1, and the 20-yr group was transitional between 10-yr and Mature meadow groups (Fig. 4). There was little difference between two years for each stage (Fig. 4).

### Seed mass variation during vegetation succession

For the 140 species examined, seed mass per seed ranged from 0.0042 mg (*Veronica eriogyne*) to 2.8768 mg (*Thermopsis lanceolata*). There no trend in seed mass distribution along a successional gradient both in vegetation (F_2005_ = 0.890, p_2005_ = 0.447; F_2006_ = 0.724, p_2006_ = 0.539) and in soil seed bank (F_July 2005_ = 0.074, p_July 2005_ = 0.974; F_April 2006_ = 0.309, p_April 2006_ = 0.819).

### Relationship between abundance and seed mass

**Figure 6:**
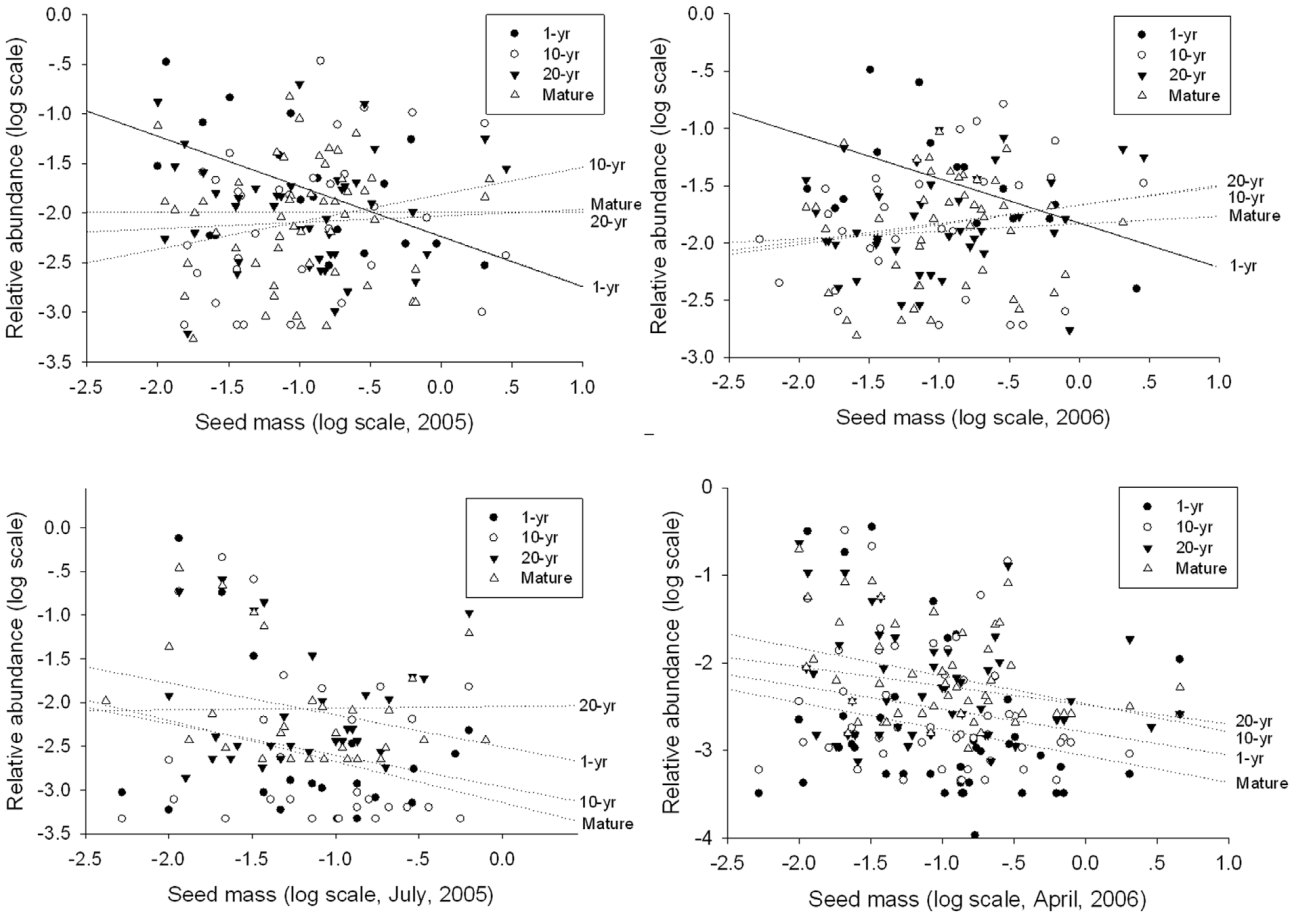
The relationship between relative abundance and seed mass during succession in the vegetation (top row) and seed bank (botton row) of a Tibetan Plateau subalpine meadow (2005 – left; 2006- right). The Kendall's tau-b correlation coefficient (r) is shown in each figure. Significant relationships (P<0.05) are denoted with solid lines.

Seed mass showed a significant negative relationship with relative abundance of vegetation in 1-yr habitat (r_2005_ = −0.401, p = 0.017; r_2006_ = −0.413, p = 0.033), but the relationships were not significant in 10-yr, 20-yr and Mature meadow habitats in both 2005 and 2006 (Fig. 6). For the seed bank, seed mass showed no relationship with relative abundance in each of four habitats in both July 2005 and April 2006 (Fig. 6).

### Relationship between seed bank and vegetation

The 1-yr vegetation group clustered together (2005) with or very close (2006) to its corresponding 1-yr seed bank group. The other vegetation groups were different from corresponding seed bank groups both in 2005 and 2006 (Fig. 4).

## Discussion

### Soil seed bank changes with succession

Species richness showed an increasing trend along the successional gradient in the shallow layer (0–2 cm) and the total (0–12 cm) for each season. This could be explained as follows. The proportion of annual species (r-selected) is higher in the early successional stage (1-yr meadow) than in later stages because of their high seed production [33]–[34] and also by their rapid growth. Seed dormancy of many of these species is a risk-spreading strategy against the temporally and spatially unpredictable environment of arable field systems [35], and is a part of an opportunistic strategy. In addition, most species, 27 (75%) species in July 2005 and 44 (67%) species in May 2006, found in the early succession stage produce long lived seeds that dominated the entire successional series although they disappeared from vegetation. As succession progressed, in mid and late successional stages, the proportion of perennial species (grasses and sedges, k-selected) (such as *Elymus dahuricus*, *Roegneria nutans*, *Kobresia humilis*, *Kobresia graminifolia*, *Kobresia capillifolia*, *Scirpus pumilus, Poa poophagorum*, *Stipa aliena*, and *Poa pratensis*) increased, and although they have low seed production they have a small input occurs to seed bank. Therefore, the species richness of the seed bank increased along the successional gradient in shallow layer and the total. [36] indicated that the seasonal fluctuations in the species composition of seed banks are due to seed losses (deep burial, predation, death, seed decay and germination) and seed gains (primary and secondary dispersal). We also found a significant difference in species richness in the two seasons (Table. 2). This is due to the presence of a transient component found only in samples collected before spring germination had occurred (May 2006) and a persistent part found in both sets of samples (May, 2006 and July, 2005). The transient part of a species seed pool germinates during spring.

Although other studies found no significant variation in seed density with time since abandonment [10], we found seed density decreased significantly along the successional gradient regardless of season (Fig. 5). This is consistent with our previous findings [5]. This decrease may be due to the interaction of several factors. First, annuals and biennials species from the early successional stage make a substantial contribution to the seed bank due to high seed production. Second, a high proportion of perennial species and colonial species (most grasses and sedges) found in later succession stages contribute little to the seed bank. The colonial species are, in rank order, *Elymus dahuricus*, *Roegneria nutans*, *Kobresia humilis*, *Thalictrum alpinum*, *Scirpus pumilus*, *Poa poophagorum*, *Stipa aliena*, and *Poa pratensis*. In addition, seeds are more easily buried because the porosity of the soil (arable activities) in the early stage of successional (1-yr meadow). Hence, during succession, the seed bank density, which is initially high, decreases.

### Relationship between species abundance and seed mass consistent along a successional gradient

Numerous studies have shown that communities of early successional stages are dominated by small-seeded species [18]–[20] that would have more colonization opportunities than large-seeded species. As succession progresses, plant communities gradually become dominated by large-seeded species in later stages [18], [20]–[21] where large seeds increase seedling survival, seedling establishment success and competitive ability [37]. A negative relationship between seed mass and abundance appears in early successional stages with a trend towards a positive relationship in mid and late successional stages [20], [27]. Therefore, it would appear that the reason for the seed mass and abundance relationship could have resulted from a competition-colonization trade-off. However, we found seed mass to be negatively correlated with relative abundance of vegetation in the early successional meadow (1-yr meadow), but the correlations were not significant and there was no trend in mid and late successional meadows (10-yr, 20-yr and Mature) (Fig. 6). The competition-colonization trade-off theory, therefore, cannot explain our results. In these subalpine meadows, the relationship appears determined by a complicated interaction between small and larger seeded species and their environment.

As known, neutral theory emphasizes the role of stochastic events, such as dispersal, and local extinction and speciation, in determining community structure, while the niche theory emphasizes the role of deterministic events, including trait differences among species, which often show trade-offs [20]. Previous research has found that a species dispersal ability is strongly correlated with its seed mass [18]–[19], [38]. Both stochastic and niche processes dominate in the early stage of succession, while niche processes dominate in later successional stages [19]–[20], [27], [39]. Our results, showing that small-seeded species were much more abundant than large-seeded species in the early stage, indicated that both stochastic processes (dispersal processes) and deterministic processes (trade off between seed mass and abundance) were important determinants of early successional structure. However, there was no relationship between seed mass and abundance in mid and later succession stages. According to our results, we could not show that stochastic processes or deterministic processes dominated in mid and later successional stages. We think change from a negative relationship in early succession to a positive relationship in later stages may appear if the late/final successional stage were dominated by shrubs or forest.

Seed mass showed no relationship to relative seed bank abundance in any of the four stages (Fig. 6). This could be attributed to a seed bank composed mainly of persistent seeds of early successional species during the whole successional range. Mid and late successional stage species may have larger seeds but they contribute little to the seed bank. [40] found that the range of seed size was relatively small in Tibetan Plateau compared with other studies. In addition, the predation pressure reducing soil survivorship in larger seeds [29].

### Potential contribution of soil seed bank during subalpine plant succession, and implication for restoration

The difference in species composition between the seed bank and vegetation could be attributed to contrasting reproductive strategies and seed persistence of annual and perennial life forms [9]. Moreover, there appears to be little potential for seed banks in grassland restoration [41]–[43]. Although [7] stated that seed banks of abandoned former grasslands could not realistically contribute to restoration of target communities, there are differences among ecosystems. [13] found that the clonal growth is important in maintaining the vegetation structure in subarctic and arctic habitats. However, [44] found that while abandoned semi-arid old-field species composition changed with succession, those present in the seed bank were similar to those in vegetation, with the trajectory of vegetation and seed bank toward to a same termination (typical grassland).

In contrast, we found the highest similarity in the 1-yr meadow and that the similarity decreased with succession (Fig. 4). These results are similar to those of many other studies [5], [45]–[46]. It is indicated that the high contribution of both the transient (May, 2006) and persistent (July, 2005) seed bank to the vegetation in early succession, and the role of seed bank decreased during succession. The important role in early succession may be related to the presence of many regeneration niches. As succession progresses, the proportion of perennial, k-selected species increases. [5], [28] found that low similarity is often a characteristic of perennial dominated grasslands. Hence, species in late succession contribute little to the seed bank, and the seed bank has little role in vegetation regeneration because there few regeneration niches.

The biodiversity and sustainable economic and social development in subalpine meadows of the Tibetan Plateau have been threatened by agricultural exploitation and overgrazing in recent years [28]. Hence, research on how to restore and manage degraded meadows is a matter of great urgency [5], [27]–[28]. Seed regeneration is one of the most important ways to achieve natural grassland restoration [28]. If the seed resources of typical grassland species in the seed bank are sufficient, successful grassland restoration might still be possible [14], [28]. Some studies indicated that the potential species pool (seed bank) is likely to be seriously impoverished under intensive arable cultivation and seed limited, and the lack of typical grassland species can be a considerable problem in restoration [14]. However, in the subalpine meadows of the eastern Tibetan Plateau, we found that a sufficient resource and appropriate species exist in the seed bank in early successional stages, and that they persist and could play an important role in restoration. Hence, we believe that the restoration of abandoned farming and grazing meadow to species-rich subalpine meadow in Tibetan Plateau can be successful using the soil seed bank. In addition although species composition returned to natural conditions within ten years of protection in abandoned subalpine meadows of the Tibetan Plateau [27], we found that the vegetation species composition of only the 20-yr meadow was similar to that of the Mature meadow (Fig. 3). These results indicate that at least 20 years are required for restoration of abandoned faming and grazing meadows to a natural subalpine meadow.
